# Artificial intelligence for clinical decision support in neurology

**DOI:** 10.1093/braincomms/fcaa096

**Published:** 2020-07-09

**Authors:** Mangor Pedersen, Karin Verspoor, Mark Jenkinson, Meng Law, David F Abbott, Graeme D Jackson

**Affiliations:** The Florey Institute of Neuroscience and Mental Health, The University of Melbourne, Heidelberg, VIC 3084, Australia; Department of Psychology, Auckland University of Technology (AUT), Auckland, 0627, New Zealand; School of Computing and Information Systems, The University of Melbourne, Parkville, VIC 3010, Australia; Wellcome Centre for Integrative Neuroimaging, FMRIB, Nuffield Department of Clinical Neurosciences, University of Oxford, Oxford, OX3 9DU, UK; South Australian Health and Medical Research Institute (SAHMRI), Adelaide, SA 5000, Australia; Australian Institute for Machine Learning (AIML), The University of Adelaide, Adelaide, SA 5000, Australia; Department of Radiology, Alfred Hospital, Melbourne, VIC 3181, Australia; Department of Electrical and Computer Systems Engineering, Monash University, Melbourne, VIC 3181, Australia; Department of Neuroscience, Monash School of Medicine, Nursing and Health Sciences, Melbourne, VIC 3181, Australia; The Florey Institute of Neuroscience and Mental Health, The University of Melbourne, Heidelberg, VIC 3084, Australia; Department of Medicine Austin Health, The University of Melbourne, Heidelberg, VIC 3084, Australia; The Florey Institute of Neuroscience and Mental Health, The University of Melbourne, Heidelberg, VIC 3084, Australia; Department of Medicine Austin Health, The University of Melbourne, Heidelberg, VIC 3084, Australia; Department of Neurology, Austin Health, Heidelberg, VIC 3084, Australia

**Keywords:** artificial intelligence, neurology, augmented intelligence, deep learning, ethics

## Abstract

Artificial intelligence is one of the most exciting methodological shifts in our era. It holds the potential to transform healthcare as we know it, to a system where humans and machines work together to provide better treatment for our patients. It is now clear that cutting edge artificial intelligence models in conjunction with high-quality clinical data will lead to improved prognostic and diagnostic models in neurological disease, facilitating expert-level clinical decision tools across healthcare settings. Despite the clinical promise of artificial intelligence, machine and deep-learning algorithms are not a one-size-fits-all solution for all types of clinical data and questions. In this article, we provide an overview of the core concepts of artificial intelligence, particularly contemporary deep-learning methods, to give clinician and neuroscience researchers an appreciation of how artificial intelligence can be harnessed to support clinical decisions. We clarify and emphasize the data quality and the human expertise needed to build robust clinical artificial intelligence models in neurology. As artificial intelligence is a rapidly evolving field, we take the opportunity to iterate important ethical principles to guide the field of medicine is it moves into an artificial intelligence enhanced future.

## Teaser

Artificial intelligence can transform healthcare as we know it, to a system where humans and machines work together to facilitating expert-level clinical decision tools across healthcare settings. We review contemporary deep-learning methods and ethical principles in artificial intelligence, as we are moving into the future of precision medicine.

## Graphical Abstract

**Graphical Abstract:**
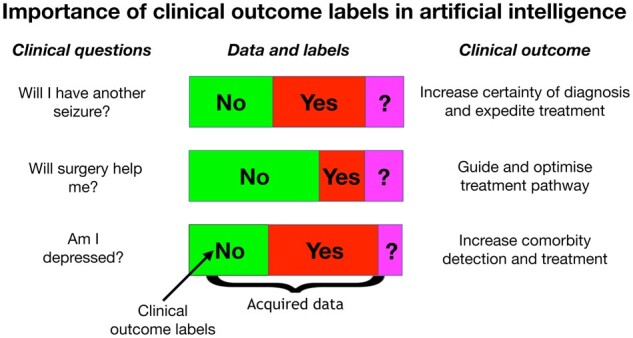

## Background—AI emulates human intelligence, processed by computer programs

**Figure 1:**
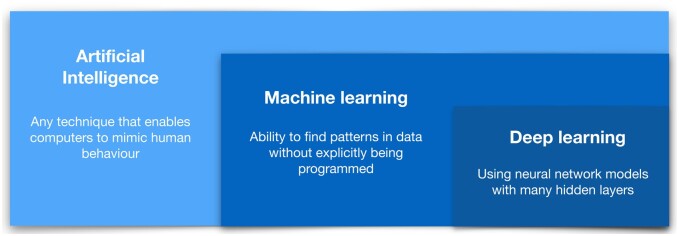
**Definitions of AI:** AI encompasses both ‘traditional’ machine learning and ‘contemporary’ deep-learning concepts.

The history of AI stems back to the 1950s with the introduction of the perceptron model (Rosenblatt, 1958; Minsky *et al.*, 2017); however, it was not until the 1990s that machine-learning techniques became more widely utilized (Crevier, 1993). The development of machine-learning tools including support vector machine and recurrent neural networks (Sarle, 1994; Cortes and Vapnik, 1995; Kohavi, 1995) allowed scientists to leverage the computational power available in this era to build statistical models robust to data variation, and to make new inferences about real-world problems (Obermeyer and Emanuel, 2016). However, arguably the biggest advances in AI to date have come in the last decade, as massive scale data and hardware suitable to process these data have become available, and sophisticated deep-learning methods—*that aim to imitate the working of the human brain in processing data*—became computationally feasible (Ngiam *et al.*, 2011; LeCun *et al.*, 2015; Schmidhuber, 2015; Goodfellow *et al.*, 2016)*.* Deep learning is now widely regarded as the foundation of contemporary AI (Sejnowski, 2020) (Fig. 1 and Box 1).

In medicine, AI has been most successfully used for image classification and prediction including detecting lung cancer and stroke based on computed tomography scans (Zhou *et al.*, 2002; Lee *et al.*, 2017; Chilamkurthy *et al.*, 2018; Zhu *et al.*, 2018; Ardila *et al.*, 2019), assessing the risk of sudden cardiac death and other severe heart diseases based on electrocardiograms and cardiac MRI (Rahhal *et al.*, 2016; Zhang *et al.*, 2017; Faust *et al.*, 2018; Hannun *et al.*, 2019) and classifying abnormal skin lesions based on dermatological images (Jafari *et al.*, 2016; Premaladha and Ravichandran, 2016; Codella *et al.*, 2017; Esteva *et al.*, 2017).

There are preliminary examples of the value of AI in neurology, for example in detecting structural brain lesions on MRI (Brosch *et al.*, 2014; Korfiatis *et al.*, 2016; Akkus *et al.*, 2017; Zaharchuk *et al.*, 2018). A common limitation of clinical AI studies is the amount of available data with high-quality clinical outcome labels, rather the availability of robust AI algorithms and computational resources. AI and deep learning are a framework that can potentially answer many disease-related questions through application of existing complex and comprehensive model architectures, so long as training data of sufficient quantity and quality is available (Box 2).

## Deep learning to extract high-level information from large and complex data

There exist several deep neural network architectures including deep neural networks, deep belief networks, recurrent neural networks and convolutional neural networks (see Sainath *et al.*, 2015). There are also methods such as Generative Adversarial Network approaches, which utilize a pair of generator and discriminator networks to improve performance (Xing *et al.*, 2019). All of these networks can learn information from large and unstructured data such as images and words, including modelling non-linear and high-dimensional features. They circumvent several limitations that have hampered efforts to translate conventional machine-learning approaches into medical biomarker discovery tools over the last decades (Ngiam *et al.*, 2011; LeCun *et al.*, 2015; Schmidhuber, 2015; Goodfellow *et al.*, 2016).

**Figure 2:**
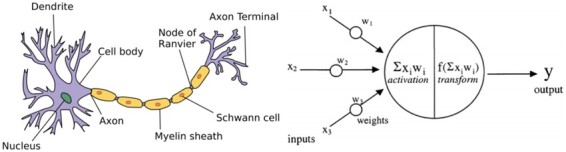
**Biological and artificial neuron:** on the left side of the figure is a biological neuron (reused under the terms of Creative Commons Attribution Licence—CC BY-SA 3.0—allowing for reproduction https://commons.wikimedia.org/wiki/ File: Neuron.svg), and on the right side of the figure is a model of an artificial neuron [reprinted from Agatonovic-Kustrin and Beresford (2000) with permission from Elsevier].

In short, deep learning deals with, and leverages, vast amounts of information whereas traditional machine-learning methods require human intervention to reduce the size of data using various feature reduction and feature selection techniques (Mwangi *et al.*, 2014; Hestness *et al.*, 2017). An intuitive way to appreciate how deep-learning works comes from understanding the firing patterns of a neuron in the brain (Savage, 2019). A neuron in the brain, as well as a node within a deep-learning network, receives inputs that they transform to an output according to a set of predefined rules that aids learning (Fig. 2;  LeCun *et al.*, 2015; Daubechies *et al.*, 2019).

**Figure 3:**
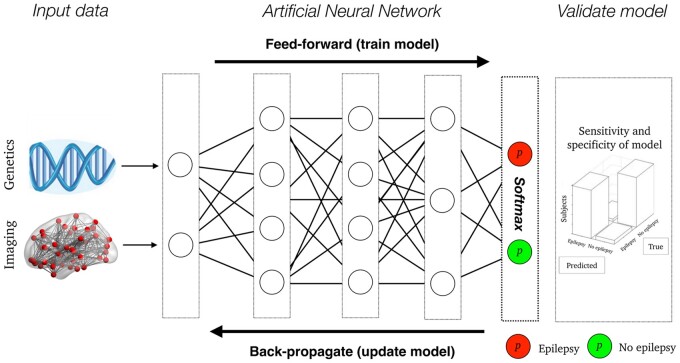
**An Artificial Neural Network example:** here is a schematic overview of how high-dimensional genetics and brain imaging is used in a deep-learning model to make a probabilistic estimate (p) whether people are likely to develop epilepsy (red node) or not (green node). The lines between layers represent connections, each associated with a weight-adjusted during feed-forward training and updated during back-propagation until the optimal model performance.

The similarity between neuronal function and AI is the reason why a deep-learning network is often called an *artificial neural network* (see Hassoun and Hassoun, 1995; Dreiseitl and Ohno-Machado, 2002 and Box 3). The sheer complexity of the brain, and deep-learning networks, arises from the interaction between multiple neurons in the brain, or multiple nodes in a deep-learning network, and how complex network interactions between multiple entities result in iterative learning. A deep-learning network learns by propagating information between multiple ‘hidden network layers’ (see Fig. 3, for a schematic overview). The hidden network layers comprise a non-linear transformation of the received input, and non-linearities make for very flexible transformations of the input data—i.e. a deep-learning neural network can ‘self-learn’ higher-order features from the input data.

To describe this process in more detail, the values of single nodes in a deep-learning model is the sum of all incoming nodes—*analogous to dendrites of a neuron—*multiplied by incoming edges—*analogous to synaptic connections*—with an added bias score—*analogous a threshold for activity (action potential) as a neuron's resting membrane potential would be*. This score is then entered into a non-linear activation function—*analogous to a neuron’s membrane potential and the threshold required to generate an action potential*. The most common activation function in contemporary AI is the rectified linear unit, a simple, fast and robust non-linear function, enabling learning within layers (Dahl *et al.*, 2013). The reason why this function is similar to initiation of an action potential (or lack thereof) is that it turns negative input values into a score of zero—*activation is not passed onto the subsequent layer*—and for positive values, its gradient is always equal to its input—*activation is passed onto the subsequent layer*. Unlike the hidden layers, the output layer of a deep-learning network has a different activation function, usually Softmax (Gibbs, 2010)*.* Softmax is popular as it provides a score across multiple output nodes with a sum of one. This means that a Softmax provides a probabilistic output that is ideal to use for prediction analysis between the deep-learning output and clinical labels of interest.

The performance of a deep-learning network is directed by a loss function that measures how accurate the output of the network is to the true clinical label value provided in the training data. There are various loss functions available including mean squared error loss, hinge loss and cross-entropy loss (Janocha and Czarnecki, 2017), all quantifying model performance in different ways, with the potential to up-weight or down-weight certain errors—allowing the trade-off between false positives and false negatives to be adjusted to the particular situation.

Once a loss function is chosen, the network learns how to perform the task by adjusting the weights between the neurons in the different layers to minimize the numerical value of the loss function over all the training examples. This is done using the back-propagation algorithm (Rojas, 1996), which determines the impact of each weight on the outcome and makes fine adjustments achieved by multiplying a pre-specified learning rate coefficient, usually a value in the range of 0.1–0.5, to the weights for each batch of training examples to improve the value of the loss function (Le *et al.*, 2011). A low learning rate value provides a smooth gradient descent of the loss function across training examples and enables detection of robust local minima—the optimal point—of the loss function (Smith *et al.*, 2018). Smith *et al.* raise a relevant point that researchers should not be tempted to increase learning rate in deep-learning model (i.e. >0.6). Higher learning rate provides faster *but less reliable* deep-learning prediction, as the local minimum is hard to find in a noisy gradient descent curve. A more reliable way to increase learning speed is to increase the batch size (the number of training examples utilized in one iteration of the deep-learning model).

## Increase AI model prediction with multimodal data

There is evidence showing that including multiple data modalities into a single AI model can result in improved model performance and predictive accuracy [see Baltrušaitis *et al.* (2017) for a review]. The scientific proposition of combining several sources of data into a single AI model remains an active field of research due to the challenge of integrating data of varying dimensionality, time scales and scope, but progress is evident as ensemble methods that take advantage of collections of separately learned models have been shown to have consistently higher performance than a single monolithic model (D’Mello and Westlund, 2015).

**Figure 4:**
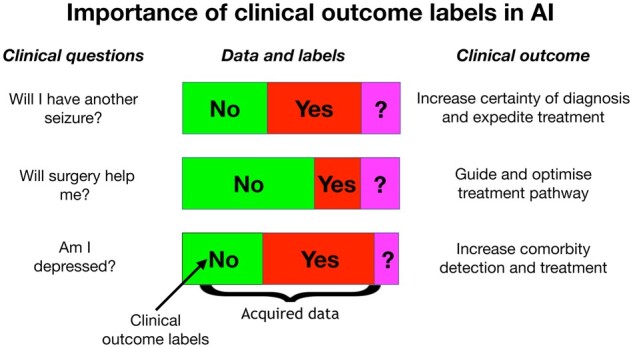
**Importance of labels in AI:** AI can answer difficult clinical questions in neurology.

An example where multimodal data are likely to be clinically effective is in epilepsy. High-dimensional brain imaging and genetics data are two types of data that have significantly enhanced our understanding of epilepsy over the last decades (Jackson, 1994; Kuzniecky *et al.*, 1997; Scheffer and Berkovic, 1997; Marini *et al.*, 2003; Dibbens *et al.*, 2013; Pedersen *et al.*, 2015; Jackson *et al.*, 2017). Incorporating such multimodal data into a single classifier is likely to result in an improved predictive AI modelling of epilepsy than a classifier relying on only a single data type, as these data sources contain complementary information pertinent to the disease. Additional data sources, such as EEG (Hosseini *et al.*, 2020; Reuben *et al.*, 2020) and clinical documentation of patient characteristics (Cohen *et al.*, 2016), may further enrich the modelling. These data are high-dimensional (Motsinger and Ritchie, 2006), so there is a lot of information that can be hard to interpret and compute with conventional statistical methods (Friston *et al.*, 1994; Benjamini and Hochberg, 1995). By using deep learning, which is designed to deal with high-dimensional data, we can start asking questions pertinent to the diagnosis and treatment of epilepsy, questions that clinicians cannot answer with current tools (see Fig. 4).

Combining multimodal data in AI models is an active area of research (He *et al.*, 2015; Badrinarayanan *et al.*, 2017; Choi and Lee, 2019), where AI models learn inherent cross-relationships between data modalities [see also Duong *et al.* (2017) for an overview]. These approaches extract and join the most useful parts of each data modality, to improve AI model performance and prediction. For example, it is possible to perform an early fusion of data (Zeppelzauer and Schopfhauser, 2016). This requires a single deep-learning model where data modalities are correlated, and their intrinsic relationships are important contributors to the outcome. Here, the model is trained on the combined representations meaning that multiple data modalities are ‘fused’ throughout all layers of the model. Although early fusion allows for better joint feature learning, it is sensitive to missing data, which also reinforces that a focus on data quality and completeness is imperative in clinical AI. Another way of combining data modalities is a late fusion of data (Cui *et al.*, 2010). This approach also requires one AI model but the assumption here is that data modalities are not significantly correlated, but their combined contribution is an important factor of the model outcome and accuracy*.* A newer model fusion technique is joint fusion (Duong *et al.*, 2017) that incorporates data at different levels of the deep-learning model. This can work well for data of different sizes including text and images.

## Validate AI models on previously unseen data by splitting data into train, test and validate sets

Any unimodal or multimodal dataset used for AI modelling needs to be divided into three different sub-categories, to ensure that we validate AI models based on unseen data (Kohavi, 1995). The data-splitting framework in AI consists of training data used to fit the AI model; testing data where the final accuracy and validity of the model is tested (Xu and Goodacre, 2018); and validation/development data separate from the training data instances enabling us to validate the model performance and tune parameters of the AI model (Ripley, 1996).

According to Liu and Cocea (2017), between 60–80% of the data is often employed to train an AI model and 20–40% of data used for testing. To fine-tune AI models and their hyper-parameters, it is important to avoid overlap contamination between training and testing data, to ensure that the AI model is tested with unseen and independent test data. It is advisable to withhold 10–30% of the training data as a validation/development dataset. The validation dataset is used to tune and optimize hyper-parameters of the AI model as this ensures that data leakage between training and test data does not occur, and therefore ensuring unbiased estimates of AI performance that are more likely to generalize to other datasets. The desired outcome of an AI model is to generate a good data-fit which is a model that resembles the underlying data. A well-fitted model also produces more accurate predictions about new data (Everitt and Skrondal, 2002; Goodfellow *et al.*, 2016). There are fallacies in model fitting that are important to be aware of and to avoid in AI analyses. A model may fit the training data ‘too well’, leading to overfitting. This overfitting often occurs in homogenous datasets, and although resulting in a valid model, it is unlikely that such a model would be generalizable (Hawkins, 2004). A model that underfits the data has not learned the patterns in the data well enough; this is usually caused by insufficient sample size. An essential requirement to avoid problems with model fitting is to obtain sufficiently large, and diverse, datasets.

## Transfer learning: previous AI models can be used as the starting point for new AI models

Transfer learning enables researchers to leverage the wealth of knowledge stored in the large and rich dataset to pre-train other AI models with (more limited) data, as this can solve other related problems or adapt to the characteristics of local data acquisition methods and demographics (Dai *et al.*, 2009; Torrey and Shavlik, 2010; Weiss *et al.*, 2016; Tan *et al.*, 2018). Transfer learning may become an important part of AI-based neurology as we want to avoid re-developing models from scratch for all diagnostic and prognostic problems that clinicians face (Kouw and Loog, 2019). An effective transfer learning paradigm will support generalization of an AI model to different populations. Predictive AI models can be altered to the local context with a significantly smaller amount of data than that required to train a model from scratch.

A successful example of transfer learning comes from a study by Eitel *et al.* (2019) who wanted to develop a diagnostic deep-learning model based on structural MRI data from a small sample of 76 people with Multiple Sclerosis and 71 healthy control subjects. This number of subjects was insufficient to train a robust deep-learning model from scratch, so the authors deployed transfer learning to pre-train an AI model based on a previously acquired, and openly released, dataset that containing 921 subjects from the Alzheimer’s Disease Neuroimaging Initiative (Petersen *et al.*, 2010). With ‘help’ from pre-trained Alzheimer’s disease data, Eitel and others were able to use transfer learning to classify people with Multiple Sclerosis from healthy control subjects with over 87% accuracy, providing a potential diagnostic test of Multiple Sclerosis based on their limited MRI data. This showcases how one can leverage large datasets and transfer learning for purposes well beyond the primary reason for acquiring the original data.

Domain adaptation also offers promising ways to improve generalizability and leverage large-related datasets to train networks (Kouw and Loog, 2019). They can also adapt the network to work better on different data—e.g. MRI scans with different quality/resolutions, or different scanners, or from under-represented patient groups. A degree of adaptation is possible even in the extreme case where no training labels are available in the new dataset, by comparing unlabelled data in the new context to the original dataset. This can be important for generalizing, or harmonizing, the network to work with data from different hospitals, using different scanners for example, where there may be insufficient data to perform transfer learning.

## Augmented Intelligence: the interplay between human expertise and AI algorithms

Although AI has the potential to transform healthcare as we know it, its success will depend on how successful we are at developing a symbiotic relationship between human domain-specific expertise and predictive AI algorithms, also optimized and fine-tuned by human experts. The concept of *Augmented Intelligence* emphasizes the assistive role of AI in advancing human capabilities and decision-making [see Gennatas *et al.* (2020) for more information]. An AI programme can provide a decision or prediction after learning patterns from data, but the interpretation and real-world implementation of AI models requires human expertise. Humans ultimately must decide how AI models should be integrated into clinical practice (Bærøe *et al.*, 2020; Reddy *et al.*, 2020).

Furthermore, understanding of the decisions made by complex AI models is a critical element of confidence in the advice they provide (Israelsen and Ahmed, 2019). This builds on trust in the models (‘AI assurance’), and being able to explain the decisions that they make (‘explainability’)—distinguishing here between explaining *decisions* and explaining the *mechanisms* by which they arrive at those decisions (Adadi and Berrada, 2018; Guidotti *et al.*, 2018; Miller, 2019). The advantage of adhering to the concept of Augmented Intelligence in a clinical and research setting is that human experts can use less time on automatable tasks such as identifying abnormal imaging features and focus on the tasks that demand uniquely human skills, including asking contextually appropriate questions about a patient’s condition, interpreting and critically analysing data, and discussing individual needs and values that may determine the best treatment decision for a given patient. Human experts may do better at understanding unusual and rare cases with uncommon pathologies, where it is not possible to get adequate training data for AI analysis—this is something that makes Augmented Intelligence important now and in the future.

The performance of an AI model must be benchmarked against a known clinical outcome that provides an appropriate target label for AI prediction (e.g. seizure versus no seizure; drug response versus no drug response; depression versus no depression). Accurate identification of these target labels requires clinical knowledge, and we are dependent on people with extensive clinical experience and expertise to provide reliable outcome measures in our patients. Humans and machines need to work *together* to ensure that the outputs of AI models are robust enough for clinical prediction (Elshafeey *et al.*, 2019).

In terms of identifying and prioritizing the problems and questions where AI methods can be most useful, the clinicians may assist in monitoring the use of algorithms in particular clinical situations—to understand at some level what the limitations of the algorithms might be, and to flag when a decision does not seem to be correct (either because it does not align with a subjective clinical intuition, or when a patient outcome is contrary to a prediction) to support further refinement and improvement of algorithms and general safety monitoring of the algorithms in practice. A common scenario in the AI community is that different research groups—with different AI algorithms—compete to produce the best predictive result to a specific clinical problem or question. This competition or crowd-sourcing approach is embodied in platforms such as Kaggle, supported by Google (www.kaggle.com). Here, researchers explore and build predictive models in a web-based data-science environment. This encourages collaboration between researchers and engineers to solve intricate data-science problems or questions that can be fed back to the clinicians for further refinement or implementation.

## AI to assist prognosis avoids potential overdiagnosis

Improvements in the sensitivity of diagnostic technology, whether or not driven by AI, have the potential to result in overdiagnosis. A classic example is the availability in South Korea of an inexpensive yet sensitive test for the presence of thyroid cancers. Its introduction and popularity resulted in an order of magnitude increase in the detection rate of thyroid cancers over a decade, entirely attributable to the detection of papillary thyroid cancer—yet over the same period, there was virtually no change in mortality (Ahn *et al.*, 2014). The ‘improved’ testing was essentially detecting an increase in benign cases, resulting in unnecessary treatment and anxiety, and wasting precious healthcare dollars. AI predictive tools trained on patient outcome measures, rather than diagnostic surrogates, prospectively avoids this problem. An outcomes-trained predictive tool provides clinicians and patients with the prognostic information they really need—for example helping to answer questions such as those indicated in Fig. 4.

## Ethical principles are imperative in the fast-changing field of AI

At present, the rapid advances in precision medicine technologies, large data and AI-led analysis are outstripping societal and regulatory response. As the pace of AI technology continues to drive transformation in health, it is imperative to consider the ethical and safety implications of AI systems for research and practice. As AI pushes the boundaries of what we can do with data, we face a responsibility to ensure that the highest standards for data management and AI development are upheld, while also ensuring the continuing development of AI tools to improve diagnosis and treatment of disease (Topol, 2019).

Public trust and confidence in AI are crucial to its success in medicine. Recent ethical frameworks promote understanding of AI ethics and regulations in medicine (Bryson and Winfield, 2017; Floridi *et al.*, 2018; Jobin *et al.*, 2019), including the Royal Australian and New Zealand College of Radiologists and the EU’s initiative to develop a trustworthy ethical framework (see Box 4).

The US Food and Drug Administration has also called on AI researchers to provide expert input on how to ensure sound governance and ethical responsibility in the field of AI in medicine (https://www.fda.gov/files/medical%20devices/published/US-FDA-Artificial-Intelligence-and-Machine-Learning-Discussion-Paper.pdf). They have proposed a set of rules intended to provide regulatory oversights of AI algorithms used in healthcare. For example, there is a low risk of using AI if its purpose is to inform clinical management in non-critical healthcare situations. But AI algorithms are of high risk when they are a driver of clinical decision-making in acute disease. Requirements for AI-based software will need to: carefully review of the safety and effectiveness of such software; address the allowable post-approval modifications to the software; and manage unanticipated divergence in the software’s eventual performance from the original product which was approved (Hwang *et al.*, 2019). Regulatory agencies, institutions and industries will need to formulate guidelines and policies regarding the use of patient data to underpin commercialization of algorithms developed using patient data.

Despite the apprehension of how AI can be misused, the Commonwealth Scientific and Industrial Research Organisation recently released an AI roadmap and alluded to the point that we need to build trust in the field of AI (https://data61.csiro.au/en/Our-Research/Our-Work). Integral to building trust in AI is quality assurance, safety, security and traceability of data and its platforms. As discussed above, AI models are superfluous without human expertise to tune and clinically interpret AI results—and clinicians and scientists need to come together to build interpretable AI models, to improve treatment and care in neurology. Ethical, privacy and security considerations are paramount in any advance of precision medicine and the use of large data sets and AI. These concerns, however, can be managed and should not lead to inertia as AI has the potential to change lives (Topol, 2019).

## Concluding remarks: large-scale projects are needed to unlock AI’s clinical potential

Precision medicine and AI is likely to be a big part of the future of medical practice (Collins and Varmus, 2015). AI has the potential to create a paradigm shift in the diagnosis, treatment, prediction and economics of neurological disease. People living with a neurological disease yearn for such precision—Will I have another seizure? Will this medication work for me? Should I have surgery? Am I depressed? Advancements in AI technology have the potential to reduce the uncertainty surrounding diagnosis and treatment of all neurological disease. But to achieve this, a deep effort is needed to fund large-scale studies with data derived from realistic clinical documentation that includes participant outcome measures. This will create an invaluable asset to drive advances in the future of healthcare.

## Abbreviations

AI: = artificial intelligence
